# *Trans*-Cinnamic Acid Stimulates White Fat Browning and Activates Brown Adipocytes

**DOI:** 10.3390/nu11030577

**Published:** 2019-03-08

**Authors:** Nam Hyeon Kang, Sulagna Mukherjee, Jong Won Yun

**Affiliations:** Department of Biotechnology, Daegu University, Gyeongsan, Gyeongbuk 38453, Korea; knhmen@naver.com (N.H.K.); m.newsulagna@gmail.com (S.M.)

**Keywords:** brown adipocyte, fat browning, *trans*-cinnamic acid, white adipocytes, anti-obesity

## Abstract

Recently, pharmacological activation of brown fat and induction of white fat browning (beiging) have been considered promising strategies to treat obesity. To search for natural products that could stimulate the process of browning in adipocytes, we evaluated the activity of *trans*-cinnamic acid (*t*CA), a class of cinnamon from the bark of *Cinnamomum cassia*, by determining genetic expression using real time reverse transcription polymerase chain reaction (RT-PCR) and protein expression by immunoblot analysis for thermogenic and fat metabolizing markers. In our study *t*CA induced brown like-phenotype in 3T3-L1 white adipocytes and activated HIB1B brown adipocytes. *t*CA increased protein content of brown-fat-specific markers (UCP1, PRDM16, and PGC-1α) and expression levels of beige-fat-specific genes (*Cd137*, *Cidea*, *Cited1*, *Tbx1*, and *Tmen26*) in 3T3-L1 white adipocytes, as well as brown-fat-specific genes (*Lhx8*, *Ppargc1*, *Prdm16*, *Ucp1*, and *Zic1*) in HIB1B brown adipocytes. Furthermore, *t*CA reduced expression of key adipogenic transcription factors C/EBPα and PPARγ in white adipocytes, but enhanced their expressions in brown adipocytes. In addition, *t*CA upregulates lipid catabolism. Moreover, mechanistic study revealed that *t*CA induced browning in white adipocytes by activating the β3-AR and AMPK signaling pathways. *t*CA can induce browning, increase fat oxidation, reduce adipogenesis and lipogenesis in 3T3-L1 adipocytes, and activate HIB1B adipocytes, suggesting its potential to treat obesity.

## 1. Introduction

Obesity is associated with numerous other metabolic complications including diabetes, hypertension, hyperlipidemia, atherosclerosis, and cardiovascular diseases [1]. Notably, obesity is caused by oversupply of energy provided by excess fat that is accumulated in the body without being consumed [2]. Besides exercise and calorie restriction, another alternative way to lose weight and reduce obesity is to increase energy expenditure by activating brown adipocytes [3].

There are three types of fat in humans: (1) white adipose tissue (WAT), which makes up nearly all fat in adults; (2) brown adipose tissue (BAT), which is involved in energy expenditure; and (3) brown in white fat (brite or beige fat), which converts from white adipocytes to brown-like adipocytes and contributes to energy expenditure in humans [4]. Targeting adipose tissue has potential therapeutic importance for the treatment of obesity and other metabolic disorders [5]. Recent discovery in the process of browning, or beiging, has heightened an interest in research for exhibiting this particular process to be an efficient technique in the conquest of obesity [6,7].

The fundamental factor leading to the process of adaptive thermogenesis is governed by uncoupling protein 1 (UCP1) [8], known to be expressed in brown and beige adipocytes [9]. UCP1 releases heat as a form of energy after uncoupling the electron transport chain for energy production [10]. It plays a critical role in energy balance and metabolic regulation of cold and diet-induced thermogenesis [11,12]. Recent studies have identified ectopic expression of other hallmark proteins, such as PGC-1α and PRDM16, as novel beige-fat-specific markers [13,14]. These proteins can be targets for the identification of brown fat-activation or browning/beiging agents [15,16,17]. Among the genetic markers, *Cd137, Cited1, Tbx1,* and *Tmem26* have been reported for beige-specific markers, while genes, *Eva1, Lhx8,* and *Zic* have been specified for brown adipocytes [18,19].

Recently, advances have been made to understand the roles of pharmacological agents and dietary supplements that contribute to browning of white adipocytes [20]. To date, a variety of natural compounds have shown promise for regulating BAT activity and recruiting beige adipocytes, as well as enhancing lipolytic and catabolic potential of WAT [20,21,22,23].

Cinnamon (*Cinnamomum cassia*) is one of the most important spices used daily by many people all over the world. Cinnamon primarily contains essential oil (*Cinnamomum vernum*) and other derivatives such as cinnamaldehyde and cinnamic acid [24,25]. Among several analogs of cinnamon, *trans*-cinnamic acid (*t*CA) is known to exhibit various health-promoting properties, including anti-diabetic [26], anti-inflammatory, and anti-cancer activities [27]. Another important feature displayed by *t*CA is that it can reduce body weight of obese rats [28] by improving insulin sensitivity and blood lipids [29].

To date, little research has been done concerning regulatory roles of *t*CA in lipid metabolism, particularly in fat browning. Therefore, the objective of the present study was to examine physiological roles of *t*CA in lipid metabolism of 3T3-L1 white adipocytes and HIB1B brown adipocytes, focusing on browning.

## 2. Materials and Methods

### 2.1. Chemicals

**Figure 1:**
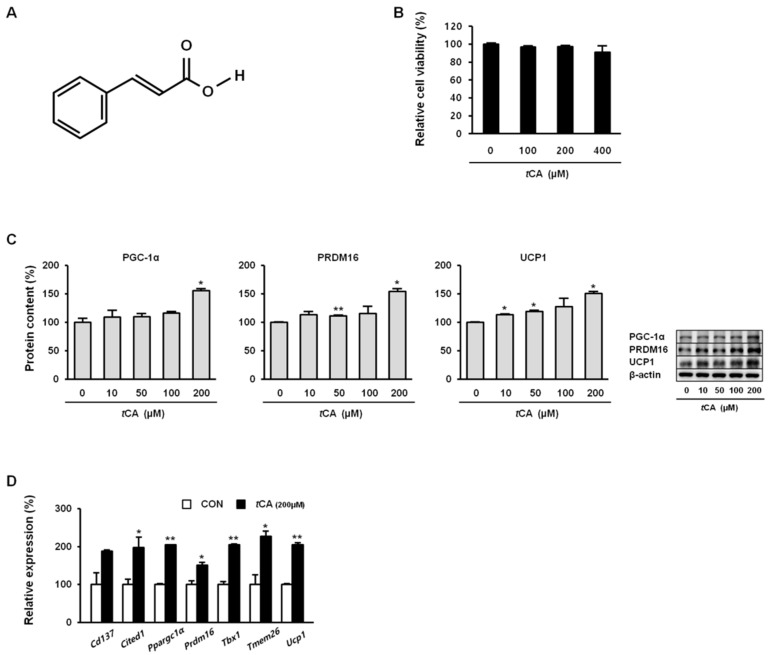
*Trans*-cinnamic acid (*t*CA) induces browning in white adipocytes. Chemical structure of *t*CA (**A**) and cytotoxicity (**B**). *t*CA promotes increased protein content of core brown fat markers (**C**) in a dose-dependent manner as well as expression of beige fat-specific genes (**D**) at 200 μM in 3T3-L1 adipocytes. Data are presented as the mean ± SD, and differences between groups were determined using the Statistical Package of Social Science (SPSS, version 17.0; SPSS Inc., Chicago, IL, USA) program, followed by Tukey’s post-hoc tests or Student’s *t*-test. Statistical significance between control and *t*CA-treated 3T3-L1 cells are shown as * *p* < 0.05 or ** *p* < 0.01.

*Trans*-cinnamic acid (99% purity, Figure 1A) was purchased from Sigma Chemical Co. (St. Louis, MO, USA). BRL 37344 and L-748.337 were purchased from Tocris Bioscience (Bristol, UK). AICAR was purchased from TCI (Chuo-ku, Tokyo, Japan). Dorsomorphin was purchased from Abcam (Cambridge, UK). All other chemicals used in this study were of analytical grade.

### 2.2. Cell Culture and Differentiation

3T3-L1 and HIB1B pre-adipocytes (ATCC, Manassas, VA, USA) were cultured in Dulbecco’s Modified Eagle’s Medium (DMEM, Thermo Fisher Scientific, Waltham, MA, USA), supplemented with 10% fetal bovine serum (FBS, PAA Laboratories, Pasching, Austria) and 100 µg/mL of penicillin-streptomycin (Invitrogen, Carlsbad, CA, USA) at 37 °C in a 5% CO_2_ incubator. Sufficiently confluent cells were maintained in differentiation induction medium consisting of 10 µg/mL of insulin (Sigma, St. Louis, MO, USA), 0.25 µM dexamethasone (Dex, Sigma), and 0.5 mM 3-isobutyl-1-methylxanthine (IBMX, Sigma) in DMEM, followed by culturing in maturation medium containing 10% FBS and 10 µg/mL of insulin. During treatments, unless otherwise stated, cells were maintained in complete medium containing 100 µM *t*CA (dissolved in dilute ethanol) for 6–8 days before further analysis. Maturation medium was changed every 2 days. Cytotoxicity of *t*CA was evaluated by MTT assay as described previously [30]. The cellular in vitro models used in this study were commercially available models. We did not use any kind of human samples to require the approval of the Ethics Committee.

### 2.3. Quantitative Real-Time Reverse Transcription Polymerase Chain Reaction (RT-PCR)

Total RNA was isolated from mature cells (4–8 days) using a total RNA isolation kit (RNA-spin, iNtRON Biotechnology, Seongnam, Korea). RNA (1 μg) was converted to cDNA using Maxime RT premix (iNtRON Biotechnology). Power SYBR Green (Roche Diagnostics Gmbh, Mannheim, Germany) was employed to quantitatively determine transcription levels of genes by quantitative RT-PCR (Stratagene 246 mix 3000p QPCR System, Agilent Technologies, Santa Clara, CA, USA). PCR reactions were run in duplicates for each sample. Transcription levels of all genes were normalized to the level of β-actin. Sequences of primer sets used in this study are listed in Table 1.

### 2.4. Oil Red O Staining

Cells were matured for 4–8 days followed by washing with phosphate-buffered saline (PBS), fixation with 10% formalin for 1 h at room temperature, and washing again three times with deionized water. A mixture of Oil Red O solution (0.6% Oil Red O dye in isopropanol) and water at a 6:4 ratio was layered onto cells for 20 min, followed by washing four times with deionized water. Images of the stained lipid droplets were visualized using an inverted microscope. Intracellular lipid content was quantified after extracting ORO bound to cells with 100% isopropanol, and absorbance at 500nm was determined in triplicate wells using a microplate reader.

### 2.5. Immunoblot Analysis

Cell lysates were prepared using RIPA buffer (Sigma) by homogenization and centrifugation at 13,000× *g* for 30 min. Cell extracts were diluted in 5X sample buffer (50 mM Tris at pH 6.8, 2% SDS, 10% glycerol, 5% β-mercaptoethanol, and 0.1% bromophenol blue) and heated at 95 °C for 5 min before 8%, 10%, or 12% SDS-polyacrylamide gel electrophoresis (PAGE). After electrophoresis, samples were transferred onto a poly vinylidene difluoride membrane (PVDF, ATTO Technology, Amherst, NY, USA) and then blocked for 1 h with TBS-T 10 mM Tris-HCl, 150 mM NaCl, and 0.1% Tween 20) containing 5% skim milk (Sigma) or BSA (Rocky Mountain Biologicals, Missoula, MT, USA). The membrane was rinsed three times consecutively with TBS-T buffer, followed by incubation at room temperature for 1 h with 1:1000 diluted primary polyclonal antibodies, including anti-ATGL, anti-ACC, anti-pACC, anti-β-actin, anti-PPARγ, anti-AMPK, anti-pAMPK, anti-UCP1, anti-PGC-1α, anti-CPT1, anti-ACOX1, anti-C/EBPα, anti-β3-AR, anti-PKA, anti-FAS (Santa Cruz Biotechnology, Santa Cruz, CA, USA), anti-PRDM16(Abcam, Cambridge, UK) and anti-pHSL (Cell Signaling Technology, Inc., Danvers, MA, USA), in TBS-T buffer containing 1% skim milk or BSA. After three washes, the membrane was incubated with horseradish peroxidase-conjugated anti-goat IgG, anti-rabbit IgG, or anti-mouse IgG secondary antibody (1:1000, Santa Cruz Biotechnology) in TBS-T buffer containing 1% skim milk or in BSA at room temperature for 1 h. Immunoblots were then developed with enhanced chemiluminescence and captured with ImageQuant LAS500 (GE Healthcare Life Sciences, Malborough, MA, USA). Every experiment was representative of three independent experiments. Protein band intensities were normalized using β-actin bands in each cell sample and band intensities were quantified using ImageJ software (NIH, Bethesda, MD, USA).

### 2.6. Immunocytochemistry

Immunocytochemistry was performed on formalin-fixed cells. These cells were incubated with anti-UCP1 (dilution 1:1000, Santa Cruz Biotechnology) primary antibody at 4 °C overnight, followed by incubation with appropriate FITC goat anti-mouse secondary antibody at room temperature for 4 h. For staining of mitochondria, MitoTracker^®^Red (1 mM, Cell Signaling Technology, Inc.) was directly added to PBB-T (PBS + 1%, BSA, and 0.1% Tween 20) at a concentration of 200 nM. Cells were then incubated at 37 °C for 2 h. After incubation, tissues were washed with PBS and subjected to immunostaining. Morphological findings were observed using a light microscope at 40× magnification.

### 2.7. Statistical Analysis

All data are presented as mean ± SD of at least three independent experiments. Statistical significance among multiple groups was determined by one-way analysis of variance (ANOVA) followed by Tukey’s post-hoc test or two-tailed Student’s *t*-test using Statistical Package of Social Science (SPSS) software version 17.0 (SPSS Inc., Chicago, IL, USA). Statistical significance was indicated as either *p* < 0.05 or *p* < 0.01.

## 3. Results

### 3.1. Trans-Cinnamic Acid (tCA) Induces Browning in 3T3-L1 White Adipocytes

First, *t*CA cytotoxicity to 3T3-L1 preadipocytes was evaluated by MTT assay. As shown in Figure 1B, *t*CA resulted in no significant cytotoxicity at concentration up to 400 μM. Hence, unless otherwise stated, cells were treated with 200 μM *t*CA to investigate its browning effect. As shown in Figure 1C, *t*CA significantly upregulated the expression of brown-fat-specific proteins PGC-1α, PRDM16, and UCP1 in a dose-dependent manner. It also significantly upregulated genes *Ppargc1a, Prdm16,* and *Ucp1* and beige-fat-specific genes *Cd137*, *Cited1*, *Tbx1*, and *Trem26* (Figure 1D).

### 3.2. tCA Activates HIB1B Brown Adipocytes

**Figure 2:**
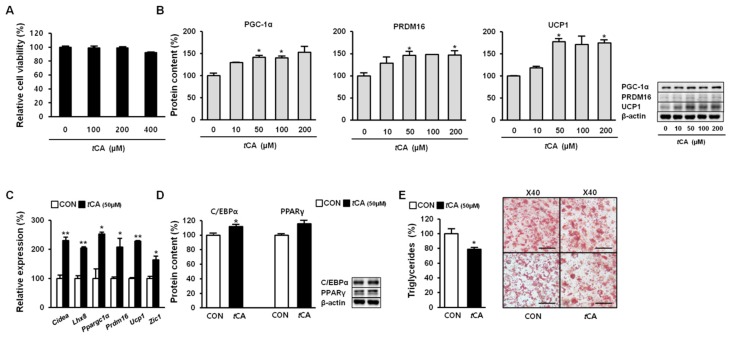
*Trans*-cinnamic acid (*t*CA) activates brown adipocytes. Cytotoxicity of *t*CA upon induction in HIB1B cells (**A**). *t*CA elevates protein content of core brown fat markers (**B**) in a dose-dependent manner as well as expression of the genes encoding beige fat-specific activity (**C**) at 50 μM in HIB1B adipocytes and regulates adipogenesis (**D**). Representative images of Oil Red O staining of HIB1B cells taken at 40× magnification (scale bars = 50 μm), where lipid content was quantified by extracting Oil Red O stain bound to cells with 100% isopropanol in brown adipocytes (**E**). Data are presented as the mean ± SD, and differences between groups were determined using the Statistical Package of Social Science (SPSS, version 17.0; SPSS Inc., Chicago, IL, USA) program, followed by Tukey’s post-hoc tests or Student’s *t*-test. Statistical significance between control and *t*CA-treated HIB1B cells are shown as * *p* < 0.05 or ** *p* < 0.01.

Since *t*CA showed no detectable cytotoxicity at concentrations up to 400 μM (Figure 2A), we further investigated whether *t*CA could activate HIB1B brown adipocytes. To this end, we allowed HIB1B adipocytes to differentiate in complete media containing different concentrations of *t*CA (0–200 μM). Our results demonstrated that *t*CA strikingly activated HIB1B brown adipocytes by enhancing expression levels of brown fat-specific proteins PGC-1α, PRDM16, and UCP1 in a dose-dependent manner (Figure 2B). It also significantly upregulated brown-fat signature genes *Cidea*, *Lhx8*, *Ppargc1a*, *Prdm16*, *Ucp1*, and *Zic1* at concentration of 50 μM (Figure 2C). Next, we determined expression levels of key adipogenic transcription factors (e.g., C/EBPα and PPARγ) in HIB1B adipocytes. Their expression levels were remarkably elevated upon *t*CA treatment (50 μM) (Figure 2D), suggesting that *t*CA could stimulate adipogenesis in brown adipocytes. In addition, *t*CA treatment (50 μM) decreased intensity of Oil Red O staining (Figure 2E).

### 3.3. tCA Promotes Mitochondrial Biogenesis in White and Brown Adipocytes

**Figure 3:**
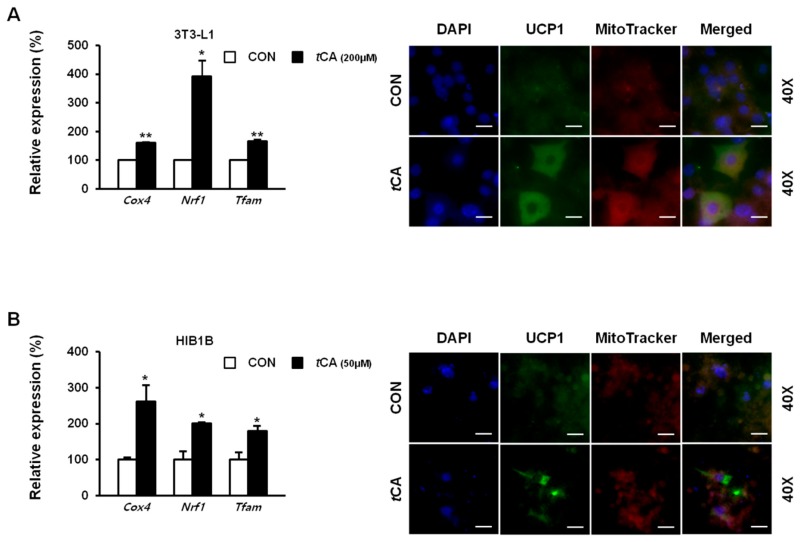
Activation of UCP1 by *trans*-cinnamic acid (*t*CA). Immunofluorescent staining of differentiated 3T3-L1 white adipocytes (scale bars = 50 μm) (**A**) and HIB1B brown adipocytes (scale bars = 20 μm) (**B**) with MitoTracker Red dye used for UCP1, when treated with *t*CA. Images were captured at 40× magnification, respectively. UCP1, uncoupling protein 1. Data are presented as mean ± S.D. Differences between groups were determined using Student’s *t*-test. Statistical significance between control and tCA-treated 3T3-L1 cells is shown as * *p* < 0.05 or ** *p* < 0.01.

As mentioned previously, *t*CA activated thermogenic marker proteins in both white and brown adipocytes. To confirm this result at genetic level we verified the mitochondrial biogenic genes *Cox4, Nrf1, MtDNA,* and *Tfam,* which expressed an elevated expression as well as at cellular level, where we directly detected UCP1 protein levels in both differentiated adipocytes using immunofluorescent staining with MitoTracker^®^Red. Results revealed stronger signals in *t*CA-treated 3T3-L1 (Figure 3A) and HIB1B (Figure 3B) adipocytes compared to those in both control adipocytes.

### 3.4. tCA Regulates Lipid Metabolism in White Adipocytes

**Figure 4:**
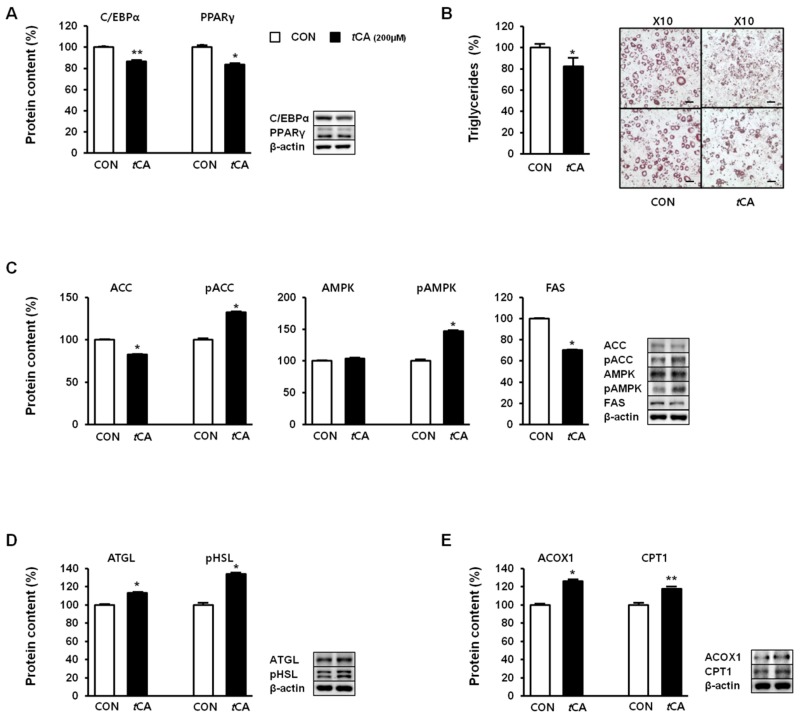
*Trans*-cinnamic acid (*t*CA) regulates lipid metabolism in white adipocytes. *t*CA regulates lipid metabolic regulators involved in adipogenesis (**A**). Representative images of Oil Red O staining of 3T3-L1 were taken at 10× magnification (scale bars = 100 μm), where lipid content was quantified by extracting Oil Red O stain bound to cells with 100% isopropanol in 3T3-L1 adipocytes (**B**), lipogenesis (**C**), lipolysis (**D**), and fatty acid oxidation (**E**). Data are presented as the mean ± SD, and differences between groups were determined using Student’s *t*-test. Statistical significance between control and *t*CA-treated 3T3-L1 cells are shown as * *p* < 0.05 or ** *p* < 0.01.

Next, we investigated the effect of *t*CA on lipid metabolism in white adipocytes. For this, we determined expression levels of key adipogenic transcription factors, such as C/EBPα and PPARγ, in white adipocytes upon induction of *t*CA in 3T3-L1 preadipocytes, before the initiation of the differentiation process. While the cells differentiated and developed into nature adipocytes, different results were observed in the adipocytes, and their expression levels were reduced upon *t*CA treatment, suggesting decreased adipogenesis (Figure 4A). Recruitment of beige cells in 3T3-L1 adipocytes consequently led to reduced fat accumulation, as evidenced by reduced triglycerides after *t*CA treatment (Figure 4B). Moreover, expression levels of acetyl-CoA carboxylase (ACC) and fatty acid synthase (FAS), as important lipogenic markers, were markedly reduced upon *t*CA treatment, along with an increased ratio of pACC to total ACC mediated by AMPK activation (Figure 4C). Next, we investigated expression levels of lipolysis-related proteins, including phosphorylated (activated) hormone-sensitive lipase (pHSL) and adipocyte triglyceride lipase (ATGL), before and after *t*CA treatment. As shown in Figure 4D, *t*CA enhanced lipolysis by increasing expression levels of pHSL and ATGL. *t*CA treatment also significantly increased mitochondrial protein levels of acyl-coenzyme A oxidase 1 (ACOX1) and carnitine palmitoyl transferase 1 (CPT1), suggesting augmented oxidative capacity upon *t*CA treatment (Figure 4E).

### 3.5. tCA Induces Browning of White Adipocytes via Activation of the β3-AR and AMPK Signaling Pathways

**Figure 5:**
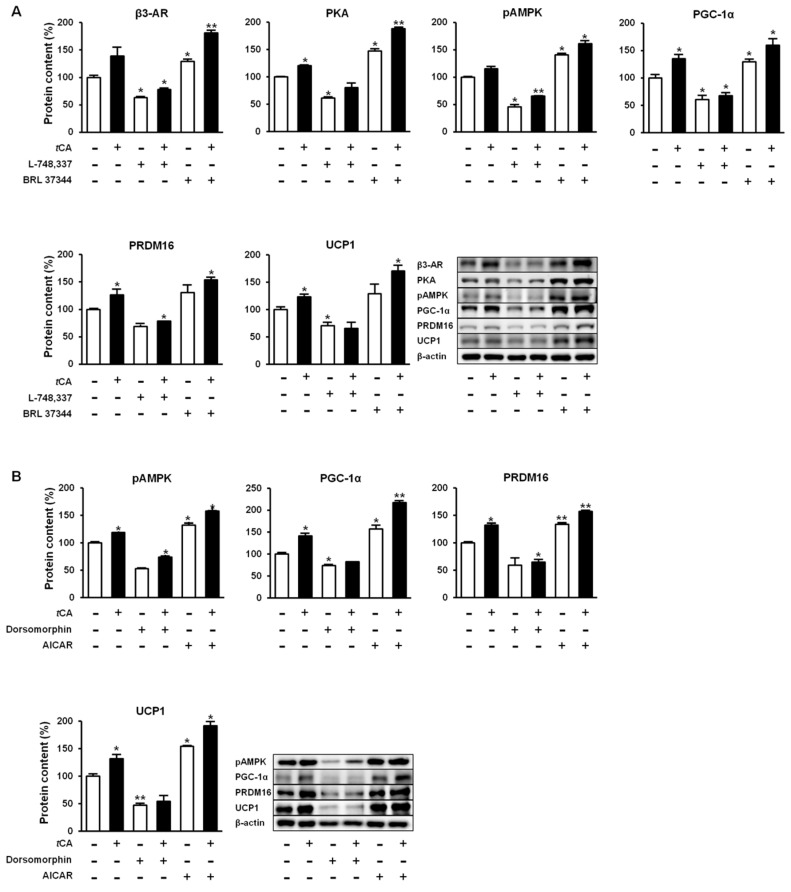
*Trans*-cinnamic acid (*t*CA) induces browning via β3-AR as well as AMPK pathway in 3T3-L1 adipocytes. *t*CA activates β3-AR and promotes browning by elevating expression of browning markers PGC-1α, PRDM16, and UCP1 (**A**) as well as AMPK-mediated activation of β3-AR to pAMPK, resulting in higher expression levels of browning markers (**B**) in comparison to the effect of β3-AR. Data are presented as the mean ± SD, and differences between groups were determined using the Statistical Package of Social Science (SPSS, version 17.0; SPSS Inc., Chicago, IL, USA) program, followed by Tukey’s post-hoc tests. Statistical significance between control and *t*CA-treated 3T3-L1 cells are shown as * *p* < 0.05 or ** *p* < 0.01.

**Figure 6:**
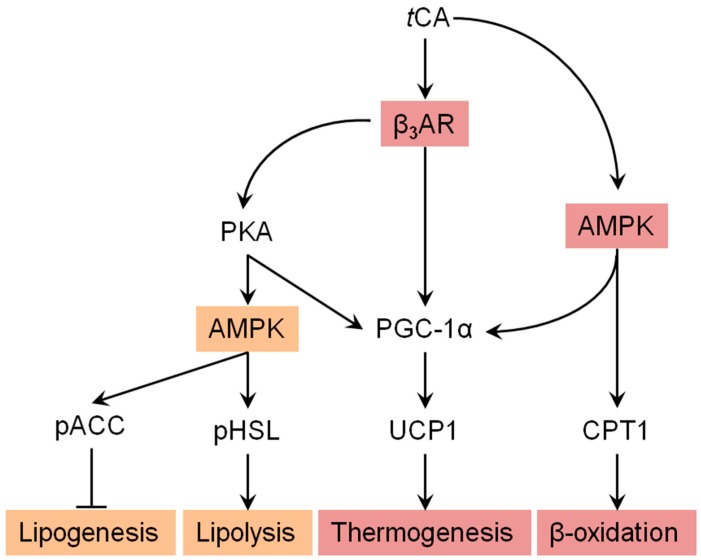
Suggested pathway for *t*CA-induced browning via AMPK-mediated β3-AR pathway. Arrow indicates stimulated regulation by *t*CA and T refers to suppressed regulation by *t*CA.

We further investigated molecular mechanisms involved in the browning activity of *t*CA. To this end, we separately treated 3T3-L1 cells with β3-adrenergic receptor (β3-AR) antagonist L-748.337 at 80 µM and β3-AR agonist BRL 37344 at 20 µM with or without *t*CA at 200 µM after 7 days of differentiation, after which expression levels of key signaling molecules (PGC-1α, PRDM16, and UCP1) responsible for browning were determined. Inhibition of β3-AR by antagonist L-748,337 resulted in reduced expression levels of PKA, pAMPK, and browning markers. It also abolished increment of β3-AR, PKA, pAMPK, and browning markers induced by *t*CA (Figure 5A). Treatment with β3-AR agonist BRL 37344 in combination with *t*CA synergistically increased expression levels of β3-AR, PKA, pAMPK, and browning marker proteins (Figure 5A). We also determined expression levels of browning marker proteins PRDM16, PGC-1α, and UCP1 after separate treatment of 3T3-L1 cells with AMPK antagonist dorsomorphin at 5 µM and AMPK agonist AICAR at 100 µM after 7 days of differentiation. Inhibited AMPK decreased expression levels of browning markers and abolished their increased levels induced by *t*CA. Browning markers were also synergistically elevated by a combination of AMPK agonist and *t*CA (Figure 5B). In addition, *tCA* elevated the expression levels up to two-fold for ATGL and p-HSL in the presence of β3-AR agonist, indicating its potential role of lipolysis mediated by β3-AR in white adipocytes (Figure S1). These results indicate that AMPK has a direct effect on browning induced by *t*CA through the β3-AR signaling pathway in 3T3-L1 white adipocytes (Figure 6).

## 4. Discussion and Conclusions

Results of the present study showed that *t*CA treatment could promote white fat browning and activate metabolic responses in 3T3-L1 white adipocytes, with a focus on the induction of beige adipocytes and elucidated the underlying molecular mechanism. Over many years, cinnamon and its derivatives have been used in traditional medicine to treat diabetes, obesity, and other metabolic diseases [29,31,32]. *t*CA is one of the active components of cinnamon, a spice produced from the bark of *Cinnamomum*. Numerous health benefits have been ascribed to cinnamon and cinnamon extract has been commercially sold to treat diabetes and other metabolic syndromes [33]. Despite a lot of reports about the beneficial roles of cinnamon and its derivatives in obesity, there is neither a consensus about bioactive constituents of cinnamon driving these effects nor molecular pathways responsible for its benefits [33,34,35]. Results obtained here contribute to the clarification of the active component in cinnamon and potential pathways involved in browning and other metabolic responses.

Recently, Kwan et al. have reported that cinnamon extract has browning effect in subcutaneous adipocytes of *db/db* and diet-induced obese mice via β3-AR signaling [36]. They have identified that components in cinnamon extract are protocatechuic acid, catechin, chlorogenic acid, and sesculetin. However, they did not specify which component was mainly involved in browning. Our data support that *t*CA might play an important role in the browning effect of cinnamon extract, although effects of other cinnamon components, such as cinnamadehyde and cinnamate, should be determined in the future. *t*CA-mediated browning also follows β-adrenergic signaling pathway through consequent activation of PKA and AMPK. However, possibility for TRPA1-agonistic action of *t*CA in browning effect cannot be excluded as cinnamaldehyde, one of the active components and 90% of the essential oil of cinnamon bark, can activate TRPA1 and increase thermogenesis [35,37]. Thermogenic activity of cinnamaldehyde needs to be determined, as it is easily oxidized to cinnamic acid. Cinnamaldehyde, an essential oil found in cinnamon, is also protective against obesity in mouse models by activating thermogenesis through the PKA-p38 MAPK signaling pathway [35,38]. Taken together, it is likely that cinnamon and its derivatives have thermogenic activity in adipocytes via the TRPA1 and/or β3AR-PKA signaling pathways.

In adipogenesis, two transcription factors, such as C/EBPα and PPARγ, tightly regulate the development of preadipocytes into mature adipocytes [39]. Suppressing these factors will reduce the accumulation of triglycerides [40]. Hsu et al. have reported that *o*-hydroxycinnamic acid can inhibit adipogenesis in 3T3-L1 adipocytes by inhibiting glycerol-3-phosphate dehydrogenase activity and down-regulating adipogenic transcription factors [41]. Similarly, *p*-hydroxycinnamic acid can suppresses adipogenesis in 3T3-L1 preadipocytes by inhibiting the MAPK/ERK signaling pathway [42]. Identical results are found with Esculetin derived from coumarin, which displayed reduced adipogenesis modulated by the AMPK pathway in 3T3-L1 adipocytes [43].

One of the important targets of AMPK is acetyl-CoA carboxylase (ACC), a key enzyme of lipogenesis, by converting acetyl-CoA to malonyl-CoA. When ACC is phosphorylated (activated), action of ACC is inhibited, thereby suppressing lipogenesis [44]. Phosphorylation of AMPK and ACC by *t*CA is related to increased mitochondrial fatty acid oxidation in adipocytes [45]. This finding is supported by increased expression of ACOX and CPT1, key players of fatty acid oxidation, upon *t*CA treatment. Work by Prabhakar and Doble and our current data support that *t*CA can reduce the expression of fatty acid synthase, thereby alleviating TG accumulation in adipocytes [46], moreover our experiments also suggested that *tCA* could decrease lipid accumulation in white adipocytes. Recent studies have demonstrated that lipogenesis and lipolysis are coupled in adipose tissue during chronic β3-AR stimulation [47]. Enhanced lipid catabolism by *t*CA is likely to be responsible for major metabolic adaptations during conversion of white to beige adipocytes [19]. Collectively, *t*CA and its derivatives could be effective compounds for improving adipocyte function.

It is well recognized that activated AMPK can switch on catabolic pathways, such as glycolysis and fatty acid oxidation, and inhibit anabolic processes, such as lipogenesis, in white adipocytes [44]. Another well-known importance of activated AMPK has been signified by inhibition of mTORC1 [48], which regulates white to beige adipogenesis, and the inhibition of mTORC1 leads to WAT browning [49]. Despite its importance in energy homeostasis, the role of AMPK in adipocyte lipolysis remains controversial. Yin and Birnbaum have demonstrated that AMPK activation is required for maximal increase in lipolysis induced by β–adrenergic stimulation [50]. In contrast, Daval et al. have argued that AMPK can block translocation of HSL to lipid droplets, thereby inhibiting lipolysis [51]. Our indirect evidence suggested that *t*CA-mediated AMPK activation could lead to stimulated lipolysis by increasing expression levels of ATGL and pHSL. The β-adrenergic signaling pathway represents a prime regulator of triglyceride breakdown by PKA-dependent phosphorylation of HSL. In the current study, *t*CA obviously activated β3-AR and consequently activated PKA, thereby phosphorylating HSL. An alternative way to activate AMPK in 3T3-L1 adipocytes has been reported by Kopp et al., demonstrating that *t*CA can activate AMPK by G-protein-coupled receptor (GPR) signaling [29].

Cold-mediated browning works practically only on beige fat depots, whereas classical brown adipocytes would be physiologically uninteresting for the browning process, as only a modest increase in UCP1 level has been detected as an effect of cold [51]. In contrast, many browning agents can induce white fat browning and activate classical brown adipocytes [52,53,54]. From this point of view, searching for agents, such as *t*CA, that can activate both white fat browning and brown fat would be a promising therapeutic strategy against obesity.

In summary, the anti-obesity effect of *t*CA was due to suppressed adipogenesis and lipogenesis as well as increased fat oxidation and enhanced thermogenesis in adipocytes, whereby the β3AR-PKA-AMPK, TRPA1, and GPR signaling pathways were responsible for thermogenic activity of *t*CA and its related components. Considering the long half-life of compounds in cinnamon [55] and good bioavailability [56] of 2.5 mmol/kg for rodents [57], consumption of *t*CA by oral administration may be a feasible way to activate thermogenesis and improve systematic lipid metabolism, thus ultimately protecting against obesity and other metabolic disorders in humans.

## Figures and Tables

**Table 1:** List of primers used for real-time quantitative RT-PCR.

Gene	Accession No.	Forward	Reverse
*Cd137*	DQ832278.1	GGTCTGTGCTTAAGACCGGG	TCTTAATAGCTGGTCCTCCCTC
*Cidea*	NM_007702.2	CGGGAATAGCCAGAGTCACC	TGTGCATCGGATGTCGTAGG
*Cited1*	NM_001276466.1	GGAAGGCACAGCACCCACTC	GGAAGGCACAGCACCCACTC
*Cox4*	NM_001293559.1	TGACGGCCTTGGACGG	CGATCAGCGTAAGTGGGGA
*Lhx8*	NM_010713.2	CATCGCTGTTCTGCCTGTTAG	CTCGGGATTCAGCAGTCCTTC
*Nrf1*	NM_010938.4	GCTAATGGCCTGGTCCAGAT	CTGCGCTGTCCGATATCCTG
*Ppargc1α*	NM_008904.2	ATGAATGCAGCGGTCTTAGC	AACAATGGCAGGGTTTGTTC
*Prdm16*	NM_027504.3	GATGGGAGATGCTGACGGAT	TGATCTGACACATGGCGAGG
*Tbx1*	NM_001285472.1	AGCGAGGCGGAAGGGA	CCTGGTGACTGTGCTGAAGT
*Tfam*	BC083084.1	ATGTGGAGCGTGCTAAAAGC	GGATAGCTACCCATGCTGCTGGAA
*Tmem26*	NM_177794.3	CCATGGAAACCAGTATTGCAGC	ATTGGTGGCTCTGTGGGATG
*Ucp1*	NM_009463.3	CCTGCCTCTCTCGGAAACAA	GTAGCGGGGTTTGATCCCAT
*Zic1*	NM_009573.3	GCCACAAATCCGGGAAGAAG	CTCACTTTCTCGCCGCTCAG

## Supplementary Materials

The following are available online at https://www.mdpi.com/2072-6643/11/3/577/s1, Figure S1: *t*CA upregulates lipolysis.

## Abbreviations

ACC	acyl-CoA carboxylase
ACO	acyl-coenzyme A oxidase 1
AMPK	AMP-activated protein kinase
AR	adrenergic receptor
ATGL	adipose triglyceride lipase
BAT	brown adipose tissue
Beige	brown in white
*Cd137*	tumor necrosis factor receptor superfamily, member 9
*Cidea*	gene encoding cell death-inducing DFFA-like effector a
*Cited1*	gene encoding Cbp/p300-interacting transactivator 1
C/EBP/*Cebp*	CCAAT/enhancer-binding protein/encoding gene
*Cox4*	cytochrome c oxidase subunit 4
CPT1	carnitine palmitoyltransferase 1
FAS	fatty acid synthase
HSL	hormone-sensitive lipase
*Lhx8*	gene encoding LIM/homeobox protein Lhx8
*NRF1*	nuclear respiratory factor 1
PGC-1α/*Ppargc1α*	peroxisome proliferator-activated receptor gamma co-activator 1-alpha/encoding gene
PKA	protein kinase A
PPAR	peroxisome proliferator-activated receptor
PRDM16/*Prdm16*	PR domain-containing 16/encoding gene
*t*CA	*trans*-cinnamic acid
*Tbx1*	gene encoding T-box protein 1
*Tfam*	transcription factor A, mitochondrial
*Tmem26*	gene encoding transmembrane protein 26
UCP1/*Ucp1*	uncoupling protein 1/encoding gene
*Zic1*	gene encoding zinc finger protein ZIC1
