# Supplementary figures and images for: Trans-Cinnamic Acid Stimulates White Fat Browning and Activates Brown Adipocytes
Source: Nutrients. 2019 Mar 8;11(3):577. doi: 10.3390/nu11030577 (PMC6470544; doi:10.3390/nu11030577)

Supplementary Materials

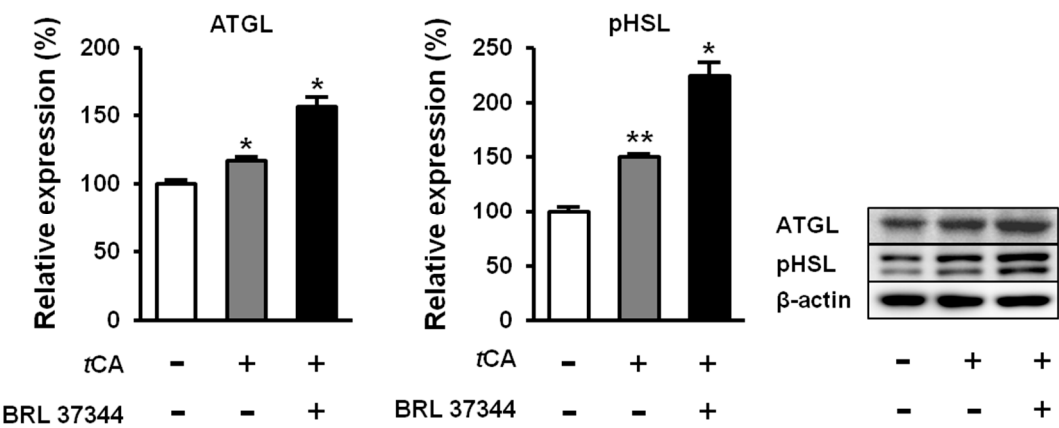

Figure 1. Figure S1: *tCA* upregulates lipolysis.

Supplement: Supplementary file 1 [file nutrients-11-00577-s001.pdf]
